# Challenges and solutions in clinical research during the COVID‐19 pandemic: A narrative review

**DOI:** 10.1002/hsr2.1482

**Published:** 2023-08-06

**Authors:** Mahin Nomali, Neda Mehrdad, Mohammad Eghbal Heidari, Aryan Ayati, Amirhossein Yadegar, Moloud Payab, Alireza Olyaeemanesh, Bagher Larijani

**Affiliations:** ^1^ Department of Epidemiology and Biostatistics, School of Public Health Tehran University of Medical Sciences Tehran Iran; ^2^ Diabetes Research Center, Endocrinology and Metabolism Clinical Sciences Institute Tehran University of Medical Sciences Tehran Iran; ^3^ Nursing and Midwifery Care Research Center, Health Management Research Institute Iran University of Medical Sciences Tehran Iran; ^4^ Students' Scientific Research Center, School of Nursing and Midwifery Tehran University of Medical Sciences Tehran Iran; ^5^ Tehran Heart Center, Cardiovascular Diseases Research Institute Tehran University of Medical Sciences Tehran Iran; ^6^ Endocrinology and Metabolism Research Center (EMRC), Vali‐Asr Hospital Tehran University of Medical Sciences Tehran Iran; ^7^ Non‐Communicable Diseases Research Center, Endocrinology and Metabolism Population Sciences Institute Tehran University of Medical Sciences Tehran Iran; ^8^ National Institute of Health Research Tehran University of Medical Sciences Tehran Iran; ^9^ Health Equity Research Center (HERC) Tehran University of Medical Sciences (TUMS) Tehran Iran; ^10^ Endocrinology and Metabolism Research Center, Endocrinology and Metabolism Clinical Sciences Institute Tehran University of Medical Sciences Tehran Iran

**Keywords:** challenges, clinical research, COVID‐19, narrative review, pandemics

## Abstract

**Background and Aims:**

The COVID‐19 pandemic has presented significant challenges to clinical research, necessitating the adoption of innovative and remote methods to conduct studies. This study aimed to investigate these challenges and propose solutions for conducting clinical research during the pandemic.

**Methods:**

A narrative review was conducted (approval ID: IR.AMS.REC.1401.029), utilizing keyword searches in PubMed and Web of Science (WOS) citation index expanded (SCI‐EXPANDED) from January 2020 to January 2023. Keywords included COVID‐19, clinical research, barriers, obstacles, facilitators and enablers.

**Results:**

Out of 2508 records retrieved, 43 studies were reviewed, providing valuable insights into the challenges and corresponding solutions for conducting clinical research during the COVID‐19 pandemic. The identified challenges were categorized into four main groups: issues related to researchers or investigators, issues related to participants and ethical concerns, administrative issues, and issues related to research implementation. To address these challenges, multiple strategies were proposed, including remote monitoring through phone or video visits, online data collection and interviews to minimize in‐person contact, development of virtual platforms for participant interaction and questionnaire completion, consideration of financial incentives, adherence to essential criteria such as inclusion and exclusion parameters, participant compensation, and risk assessment for vulnerable patients.

**Conclusion:**

The COVID‐19 pandemic has significantly impacted clinical research, requiring the adaptation and enhancement of existing research structures. Although remote methods and electronic equipment have limitations, they hold promise as effective solutions during this challenging period.

## 1 INTRODUCTION

The COVID‐19 disease has caused millions of deaths around the world. The most common symptoms of the infection were respiratory problems, which caused the disease to spread rapidly.^1^ At the beginning of the pandemic, measures such as quarantine, social distancing, rapid tracking of patients and restriction of presence in closed spaces, and crowding were carried out to control the disease.^2^ In the absence of definitive treatment and effective vaccines, these measures were effective to some extent but negatively affected society.^3^ The COVID‐19 pandemic changed many aspects of human life worldwide, causing adverse social and economic consequences that will persist for years.^4^, ^5^

Research is a critical aspect of responding to public health emergencies. Research efforts from various groups were focused on the origin of the COVID‐19 disease and management strategies, including drugs and vaccines through numerous clinical trials.^6^ Health scientists were confronting COVID‐19 and solving its complications with investigations, research, and clinical trials. Many researchers were faced with significant challenges due to the spread of COVID‐19. These issues include the unwillingness of volunteers and research participants, reductions in research funds due to shifting toward the treatment and hospitalization of the affected, and emerging difficulties in traveling and field investigations. Furthermore, stress and concerns about COVID‐19 were shared among the participants, volunteers, research team, and scientists.^7^

Field investigations are necessary for many studies, requiring the presence of scientists in the clinical environment. Furthermore, the presence of participants and volunteers is essential for many researchers. Ethical problems such as obtaining consent to participate in the research, explaining the purpose of the study, follow‐ups, and referring vulnerable patients during the study were negatively affected by the pandemic.^8^ Due to the rapid spread of the COVID‐19 disease and its high mortality, there was an urgent need for research on medications, vaccines, improving diagnostic tools, and medical management, which was a challenge for scientists.^9^, ^10^

The COVID‐19 pandemic has significantly impacted various clinical research methods, particularly clinical trials, which are crucial in evaluating the safety and efficacy of new medical treatments. Many aspects of clinical trials, including patient recruitment, obtaining informed consent, and implementing interventions, were traditionally conducted in person by the research team. However, due to the pandemic, these processes have been disrupted, and alternative methods have had to be developed to ensure the continuity of clinical research while prioritizing the safety of participants and researchers.^11^ The COVID‐19 pandemic also affected clinical research about infectious diseases as well as other medical fields, including cancer, chronic diseases, obstetrics, and gynecology.^12^, ^13^

The COVID‐19 pandemic led to the discontinuation of several significant health studies. This was primarily due to a lack of effective preparation for such a crisis and inadequate guidance for clinical researchers on addressing research challenges by utilizing various strategies rather than halting research in clinical settings.

As a result, many researchers were not equipped to adapt to the new circumstances and continue their studies safely and effectively. However, it is crucial to note that innovative solutions have emerged in response to these challenges, such as remote data collection and telemedicine, which have enabled researchers to continue their work while ensuring the safety of study participants and staff. Going forward, it is essential to prioritize preparedness for potential crises and provide clear guidance to researchers to ensure the continuity of critical health studies during challenging times.

Previous reviews have examined the challenges of conducting research during the COVID‐19 pandemic. However, these reviews are limited to clinical trials, research sponsors, or particular diseases.^7^, ^14^ A comprehensive review that examines all aspects of conducting clinical research during the pandemic is currently lacking, yet it is crucial for future crises.

Therefore, we aimed to comprehensively review the challenges faced worldwide in conducting clinical research during the COVID‐19 pandemic. We will also identify solutions to these challenges to aid researchers and policymakers in facilitating clinical research during future crises. By conducting this review, we hope to provide a comprehensive understanding of the impact of the pandemic on clinical research and contribute to the development of strategies to mitigate its effects on research activities.

## 2 METHODS

### 2.1 Study design

This study was a narrative review approved by the ethical committee of the Iranian Academy of Medical Sciences (IAMS) (REC approval ID: IR.AMS.REC.1401.029).

### 2.2 Search strategy and information sources

We searched PubMed and Web of Science (WOS) citation index expanded (SCI‐EXPANDED) on January 4, 2023, to identify related articles published between January 2020 to January 2023. We developed the search strategy for the PubMed database and modified it for the WOS database (Table 1). The search strategy was applied without any limitation on data and languages. Manual searching in key journals for relevant articles was conducted after the initial search of databases, and the reference list of included articles was checked for possible related studies (Figure S1).

**Table 1:** Search strategies used to retrieve related documents.

Search syntax in **PubMed** database
(covid‐19 OR Sars‐cov‐2 OR coronavirus OR Cov‐19 OR 2019‐ncov OR covid19 OR “covid‐19 pandemic”) AND (“clinical research” [tiab] OR “clinical study” [tiab] OR “clinical trial*“ [tiab]) AND (barriers[tiab] OR obstacles[tiab] OR challenges[tiab] OR difficulties[tiab] OR facilitators[tiab] OR enablers[tiab]) AND 2019/01/01:2023/01/01[dp]
Search syntax in **Web of Science (WOS)** citation index expanded (SCI‐EXPANDED)
(ALL = (covid‐19 OR sars‐cov‐2 OR coronavirus OR Cov‐19 OR 2019‐ncov OR covid19 OR “covid‐19 pandemic”) AND TS = (“clinical research” OR “clinical study” OR “clinical trial*“) AND TS = (barriers OR obstacles OR challenges OR difficulties OR facilitators OR enablers) AND PY = (2019‐2023))

### 2.3 Study selection process

Two authors reviewed and screened all retrieved documents independently (MN, MH) based on titles and abstracts according to the eligibility criteria. Afterward, the full texts of the selected studies were assessed. Any disagreements were resolved by the third author (NM).

The inclusion criteria consisted of studies conducted during the COVID‐19 pandemic examining challenges related to conducting research regardless of their study design. There is no limitation on the type of studies. Articles without abstract, full‐text, or sufficient relevant data were excluded.

### 2.4 Data extraction

The list of data extraction included the first author's name, publication year, study design, and study country or setting. Information about the challenges and related solutions was extracted and reviewed from the included articles narratively. To categorize challenges, we used expert opinion.

## 3 RESULTS

A total of 2508 records were retrieved through electronic databases. After removing duplicates and screening based on title and abstract, the full texts of 156 studies were assessed, and 43 studies were included in the qualitative synthesis (Figure S1).

All the studies were conducted during the COVID‐19 pandemic and evaluated the challenges of conducting research in this era. The characteristics of the included studies are provided in Table 2.

**Table 2:** Characteristics of included studies.

Author	Year	Location	Study type	Main findings
Collier, Erin K^15^	2020	USA	Editorial	Investigators must conduct clinical trials in a safe, compliant, and ethnically appropriate manner.
de Miguel, Maria^16^	2020	Spain	Editorial	Individualizing patient management while guaranteeing their safety and adherence to the study protocol, implementing specialized staff contingency plans, and maintaining sponsor and contract research organization (CRO) alignment are some of the critical problems for the research's long‐term success.
Ellen Townsend^17^	2020	United Kingdom	Editorial	To be deemed ethical, all research must have a high benefit‐to‐risk ratio.
Neeta Kantamneni^18^	2020	USA	research agenda	More research is needed on individuals experiencing increased discrimination or workload due to the pandemic.
Michelle I. Cardel^19^	2020	USA	Quantitative/qualitative cross‐sectional survey	COVID‐19 has four effects on research experiences: transition, remote intervention delivery, ability to adhere to program goals and interest in research involvement
Celeste Cagnazzo^20^	2020	Italy	Editorial	The quest to quickly uncover answers to health concerns and therapeutic ideas in the specialized clinical research domain has underlined the necessity for efficient, timely, ethically right research.
Adam Palayew^21^	2020	Canada	Editorial	Rapid publication ensures that new evidence is shared on time, particularly during a fast‐moving health crisis such as the COVID‐19 pandemic. Nonetheless, there are steps that the scientific community can and should take to prevent the accelerated pace of COVID‐19 publishing from weakening the evidence base.
Hamasaki, Toshimitsu^22^	2020	USA	Editorial	The COVID‐19 pandemic poses huge hurdles, particularly to medical research, as these disruptions have wreaked havoc on ongoing and planned clinical studies.
M Wolkewitz^23^	2020	Germany	Editorial	Numerous methodological challenges are associated with producing, gathering, analyzing, reporting, and publishing data in the compressed timeframes required during a pandemic.
Padala, Prasad^24^ R	2020	USA	Perspective	The risk‐benefit ratio of conducting, rescheduling, or canceling each research visit is determined by the principal investigator (PI). To ultimately decide on the course of action, the PI should examine the ethical principles of research, local/national advice, the community danger of the pandemic in their region, staffing strain, and the risk involved to each participant.
M Akacha^25^	2020	Switzerland	Editorial	The COVID‐19 pandemic and associated measures may also impact the types, incidence, severity, and duration of adverse events (AEs) recorded for experimental therapies and in the control group.
Sarah J Richardson^26^	2020	UK	Commentary	COVID‐19 research must be inclusive, particularly involving elderly living with frailty, cognitive impairment, or multimorbidity. Non‐COVID‐19‐related research for older people remains critical and must not be neglected in the rush to study the pandemic. Significant changes are required in designing and delivering research for older people in a world where movement and face‐to‐face contact are restricted.
Xitao Ma^14^	2020	China	Commentary	Ethical review‐related regulations must be updated, and a unified supervision system for the overall ethical review committee is required.
B.E. Bierer^11^	2020	USA	Editorial	During COVID‐19, clinical research was halted or terminated due to urgent patient care needs, and clinical trials focused on treating and preventing coronavirus infection were prioritized over studies focused on other diseases. Electronic data collection and cloud computing; And obligations to share protocols, consents, and data should be applied to rigorous research methods in the service of public health
Shahmir H Ali^27^	2020	USA	Web‐based survey	It is vital to use social media recruitment for the rapid collecting of survey data related to rapidly emerging health problems, such as COVID‐19.
Leonardo Tamariz^28^	2021	USA	Editorial	Appropriate scientific review of protocols, Devoting special research ethics committees to Covid‐19‐related research, Using alternate members and consultants, and facilitating the conduct of sound scientific and ethical research are the steps that are effective in reducing the pressure of research ethics committees.
Mohan, Sumit^29^	2021	USA	Editorial	Process flexibility for staff and research participants may be the catalyst needed to make sustainable improvements to our research processes, roles, and goals.
Shields, Charlotte N^30^	2021	USA	Survey study	The most significant barriers to follow‐up included fear of COVID‐19, wait times, and costs.
Jay J H Park^31^	2021	Canada	Editorial‐Series	The COVID‐19 pandemic has re‐emphasized the importance of well‐designed randomized clinical trials and highlighted the need for large‐scale clinical trials structured according to a master protocol in a coordinated and collaborative manner.
Caputo, Eduardo L^32^	2021	Brazil	Editorial	During the COVID‐19 pandemic, at the same time as there are changes in the format and conduct of research, the process of conducting research should not be interrupted.
Susanne Röhr^33^	2021	Germany	Perspective Study	There is a need for special statistical programs to conduct research during the COVID‐19 pandemic.
MeeLee Tom^34^	2021	USA	Perspective Study	Strong communication and constant commitment, combined with technical capabilities for remote work, visits, and study medicine distribution, were critical to the effective retention of study participants and resumption of enrolment.
Anderson, Melanie^35^	2021	USA	Perspective study	COVID‐19 has significantly impacted clinical studies, and there is a need to change the format of different phases of clinical trials.
TL Loucks^12^	2021	USA	special communication	virtual visits and digital approaches are ways to facilitate research conduction.
Perrine Janiaud^36^	2021	Switzerland	Survey	Collaborative efforts such as consortiums of trials prospectively planning to pool their results4 and adaptive platform trials such as the RECOVERY trial5 are promising approaches to provide reliable and timely evidence.
B Hensen^37^	2021	UK	Commentary	Remote data collection is one of the strategies to continue conducting public health research.
Stephanie Tremblay^38^	2021	Canada	Commentary	Conducting interviews in qualitative studies in the era of COVID‐19 is considered a fundamental challenge, and remote methods can, to some extent, continue qualitative research.
Abhinav Bassi^39^	2022	Australia	Editorial	Remote methods, the necessity of prioritizing research, and improving research infrastructures are among the critical measures in solving the challenges of conducting research.
Jenail Mobaraka^40^	2022	USA	Editorial	Conducting in‐person data collection during a pandemic would place participants and researchers at risk of infection. Therefore, adjusting and compromising the study's goals, design, and methodology to address the new subjective conditions of all actors involved are crucial protective measures.
Catherine A. Sewell^41^	2022	USA	Special Report	increased collaboration among stakeholders (federal agencies, industry, academia, and patients and patient advocates) can support progress in conducting research.
Walshe C^42^	2022	UK	Multinational Survey	Consideration must be given to widening the volunteer base away from those most vulnerable to COVID‐19.
Jon Salmanton‐García^43^	2022	Germany	Epidemiological Study	The VACCELERATE Volunteer Registry is an active single‐entry point for European residents interested in participating in COVID‐19 clinical trials.
Donna A. Santillan^44^	2022	USA	Report	Adaptability is essential for network site maintenance. Constant intra‐ and inter‐institutional contact were necessary to manage the rapidly shifting rules for starting and continuing research during the epidemic.
RM Haynes^45^	2022	Canada	Point of view	The use of technology can prevent the challenges of conducting research in the era of COVID‐19.
LA Simmons^46^	2022	USA	Study design	Remote clinical trials have the ability to not only boost representation and reduce participant travel and study visit hardship but also introduce implementation and participant retention problems.
Rashmi K. Sharma^47^	2022	USA	Editorial	During COVID‐19, the use of technology and virtual platforms is critical in doing research.
Diallo, Alpha^48^	2022	France	Commentary	Effective medical teamwork is critical in responding to epidemics/pandemics. Regulatory, legal, and financial barriers have dramatically delayed clinical trial efficiency, which is untenable during an active epidemic. Adaptive, large‐scale clinical studies during pandemics should be regarded as a key countermeasure, and regulatory approval should be expedited in accordance with the situation's urgency. This applies to non‐emergencies as well as multicenter clinical trials in general.
Daniel Munblit^9^	2022	UK	Editorial	There is an urgent need to refine and standardize outcome metrics for this significant patient group for clinical services and research, as well as to enable data comparison and pooling.
Ricardo Almeida‐Magana^49^	2022	UK	Editorial	During the COVID‐19 pandemic, remote e‐Consent‐based recruiting was vital for trial continuation.
Gianna McMillan^50^	2022	USA	Editorial	Innovative trial designs, such as basket and umbrella studies, designs that use external data sources, multi‐stage seamless trials, and preplanned data sharing amongst larger trials, are required in pandemic situations.
Micah A. Skeens^51^	2022	USA	Survey	Social media recruiting reduced traditional time and engagement hurdles for participants while also avoiding social and physical distancing requirements imposed by the pandemic, allowing for real‐time assessment of the pandemic's effects on families.
Kellie Pertl^52^	2023	USA	Cohort Study	Researchers had to move from in‐person to virtual recruitment tactics to reach and engage potential study participants during the COVID‐19 epidemic. During a pandemic, virtual recruitment looks less efficient and has hampered efforts to achieve recruitment goals.
Theresa Burgess^6^	2023	South Africa	Qualitative exploration	REC members recognized numerous substantial ethical issues and problems in examining COVID‐19‐related research. While RECs are resilient and adaptive, weariness among reviewers and REC members was a big concern.

The challenges included “issues related to researchers or investigators, issues related to participants and ethical concerns, administrative issues (i.e., research ethics committee [REC] or institutional review board [IRB] approval), and issues related to research conduction,” which are reviewed in the following sections.

### 3.1 Issues related to researchers or investigators

The increasing pressure of the pandemic on healthcare systems caused an extensive change in the employee workflow, increasing the duties of research personnel.^53^ Research nurses had to work as clinical nurses in labor, delivery, and postpartum units. The pressure of researching clinical staff was significantly reduced, allowing them to respond to the patient's needs.^39^ The research staffs were concerned about the risk of COVID‐19. Exposure to the disease during face‐to‐face meetings increased the chance of infection. Reducing the number of in‐person meetings and planning them through video conferences were necessary to reduce the risk of disease transmission. Also, appropriate personal protective equipments were mandatory to address these concerns.^7^, ^12^ Although the COVID‐19 limited medical students' classes and their presence in the hospital, many remained at their workplaces and engaged in their clinical and scientific activities.^54^ At some institutions, medical students and residents played a more prominent role in screening, consenting, and enrolling clinical research participants. Tasks that were generally performed by research personnel. For example, residents performed research activities for high‐priority research regarding public health concerns such as COVID‐19 instead of research staff.^9^ The participation of medical students in research varied in different countries and was related to the policies of each country and university.^55^ The activity of medical students may be necessary and temporary in some critical times, such as the COVID‐19 pandemic.^54^

International research and collaboration among researchers can enhance global knowledge and awareness. However, differences in goals, research priorities, and pandemic conditions can hinder cooperation. Despite these challenges, international researchers can collaborate on shared topics such as preventing disease spread, treatments, and vaccines, including different phases of clinical studies^40^ (Table 1).

### 3.2 Issues related to participants and ethical concerns

During the COVID‐19 pandemic, study participant presence was a significant research limitation due to quarantine, social distancing, travel restrictions, and participant concerns. Many participants withdrew from studies due to infection fears, while high‐risk populations, such as infants, the elderly, and pregnant women, were still needed for research purposes.^41^

Pandemic circumstances caused additional burdens on the health system, including the psychological pressure on researchers to provide a solution for the pandemic. Therefore, The process of project approvals should be revised in terms of speed, prioritization, and the presence of experts in the ethical approach. The research hypotheses may not be investigated in time if they are not approved and started at the right time. Also, prioritizing critical issues related to health in the ethics committees should be considered due to the crisis conditions.^6^ During the Ebola epidemic, for example, clinical trials proceeded non‐stop. Research design and conduction should differ from traditional approaches during infectious disease outbreaks.^7^

The main ethical challenges that organizations should investigate were obtaining informed consent and addressing ethical issues according to the study design and human interventions.^7^ Another ethical issue that organizations should consider was the participants' interest in participating in research. For any research to be considered ethical, its benefits should be higher than its risks. Moreover, COVID‐19 has psychological effects on individuals. Thus, studies on mental health, depression, suicide, and self‐harm had to be carefully considered. In high‐risk projects, the purpose of the research, the stakeholders, and how it can be implemented should be apparent. Ethics committees worldwide must consider these fundamental issues and examine them seriously.^17^, ^56^

The expansion of online methods created favorable flexibility against COVID‐19 restrictions, such as obtaining consent electronically, responding online to ethical issues, and creating a platform for employees to handle research files remotely and outside work and office hours.^7^ Some of the remote qualitative methods that were utilized included online or phone‐based interviews and focus group discussions, audio‐diary forms, photovoice (use of photography to capture lived experiences), video documenting, documentary analysis of social media (e.g., Facebook and WhatsApp groups, YouTube comments or podcasts) and auto‐ethnography. Remote quantitative methods included mobile phone surveys implemented using: interactive voice response (IVR), short messaging service (SMS), or computer‐assisted telephone interviews (CATI) and self‐completed online questionnaires shared via email or social media platforms. These methods were not new, with telephone and postal surveys used in higher‐income countries, yet their use became essential during the COVID‐19 era to support data collection directly from individuals and populations. Using technology in conducting studies was different in each country, and it depended on the national policies regarding the use of technology in health‐related research.^37^ A study by Megana found that remote e‐consent‐based recruitment was crucial for trial continuity during the COVID‐19 pandemic. This method adheres to ethical and regulatory guidelines for informed consent while minimizing face‐to‐face interactions that increase COVID‐19 transmission risk. Patients provided positive feedback on using these platforms.^49^

Ensuring participant safety and privacy are critical ethical considerations in clinical studies.^15^, ^28^ Accountability, tracking, and follow‐up before and after interventions must be prioritized to continue trials. Confidentiality of patient data and the secure delivery of investigational treatments from trial sites are essential. Participants must also be provided with instructions for properly storing and using investigational drugs.^16^, ^29^

Result by Shields et al.^30^ showed that fear of COVID‐19 was a major barrier to follow‐ups. This fear included patients who felt unsafe exposing themselves or their family members or a patient's family member feeling unsafe exposing the patient. The next most commonly reported barriers were long waiting times and financial costs.

Informed consent is a common and fundamental part of any clinical research. It is usually provided by paper forms that explain the purpose of the study, the procedures, and possible adverse effects and are signed by the participants.^28^, ^57^ A virtual electronic consent form is an alternative approach to traditional written forms.^16^ Considering the risk of infection transmission during pandemics, consent can be acquired electronically. Verbal consent for quarantined patients can be obtained first in the presence of a witness, followed by written consent when participants are released from quarantine. Thus, institutions should allocate the necessary resources to develop an appropriate consent form. Facilitating communication between participants, researchers, and institutions can help better collaboration between participants.^57^

Many studies are conducted on healthy community populations, and some projects are carried out on sensitive populations and high‐risk patients. Various studies are conducted in the suburbs and villages. These populations are essential in many ways, including dangerous risk factors such as obesity, unemployment, health considerations, and community health. However, the COVID‐19 pandemic prevented these individuals from participating in studies, and effective incentives were needed to encourage participation.^58^, ^59^ On the other hand, research on sensitive populations was considered dangerous. Addicts, sex workers, and the homeless did not follow many health protocols. Many lived in the same room with several people and did not practice social distancing. These cases could cause the spread of the coronavirus to the researchers and others, endangering the health of the participants and study operators.^18^, ^58^

Another effective way to attract participants is through financial incentives. Allocating the necessary funding for these incentives is a task that health organizations should notice. Providing essential funding, creating financial incentives, and paying attention to the participants' health can facilitate active participation in the research.^58^ A study by Basel showed that statistically significant increases were seen in participants' consent rates and responses when offered even small monetary value incentives. These findings suggest that incentives may be used to reduce the rate of recruitment failure and subsequent study termination^60^ (Table 3).

**Table 3:** Challenges and solutions in clinical research during the COVID‐19 pandemic.

Challenges		Solutions
Issues related to researchers or investigators	Risk of transmission (among research staff and investigators Reference)^61^ Increasing workload of clinical nurses^7^, ^9^, ^39^	Using remote monitoring in the form of telephone and/or video visits^61^ Reduction the number of face‐to‐face meetings and planning for video conference Using the patient's local facilities^61^ Assigning most of the research tasks to the research staff, medical students and residents^9^, ^40^
Issues related to participants and ethical concerns	Human ethics in study enrollment^5^, ^11^, ^41^ fear of infection among participants^7^, ^41^, ^50^ High risk participants (Elderly people, pregnant women)^41^, ^45^ Obtaining informed consent form^7^, ^24^, ^49^	Expansion and diversity in communication networks^6^, ^19^ Using online methods to collect information and interview^19^, ^37^ Changing the type of execution^19^ Creating online classes, online exercises, using videos and virtual networks^11^, ^56^ Creating online platforms for responding to participants, and completing questionnaires^49^, ^56^ Considering financial incentive^56^
Administrative issues	Delay in approving and reviewing documents^61^ Different and non‐specialist reviewer^16^, ^28^ Separate reviewing in each hospital and university^20^	Considering important issues such as inclusion and exclusion criteria, participant compensation, and assess the risks of studies for vulnerable patient^15^, ^29^, ^61^ Presence of experts in viral and contagious diseases in IRB^28^ Prioritizing initiated studies from a public health perspective^39^ expedite reviewing of academic trials that address important public health questions^39^ streamlining regulatory approval processes with established timelines^39^ approval of an ethics committee is accepted throughout all hospital and universities^20^
Issues related to research conduction	Low participation of volunteers in research^38^, ^43^, ^62^ Disturbance in data extraction^35^, ^36^, ^63^ Reduction of face‐to‐face visits due to the risk of contracting COVID‐19^7^, ^15^, ^25^ lack of experience regarding the use of virtual platforms^39^ statistical models and methods^22^, ^33^ funding and financial sponsorship^48^ Disturbance in follow up.^21^, ^30^ Quality of publication during COVID‐19.^31^ Refusals and losses of follow‐up^32^ Research in a special population^26^, ^47^	Accessing to epidemiological data for collecting data^25^, ^43^ Artificial intelligence and deep learning algorithm in analysis^21^, ^25^ Adapting statistical methods to the pandemic conditions^64^ Reduction the number of face‐to‐face visits^7^, ^15^, ^51^ Visits are conducted remotely or via phone or video call^7^, ^14^ Mailing drug to the participants 47 and sending the medicine directly to the patient's home by site personnel or sponsors^20^, ^63^ Using volunteer registry for enrolment of patients^18^ Making strong communication and commitment to participants^52^ Creatingtechnologicalcapabilitiesforteleworking,^34^ visits^35^, ^47^ Motivating to perform procedures at the patient's home^63^ Permitting–use, healthcare facilities,^65^

### 3.3 Administrative issues (REC or IRB approval)

#### 3.3.1 Rapid review of research

Thousands of clinical trials were registered in the first few months of the pandemic, facing ethics committees with a high load of studies. A thorough review was necessary to prevent high‐risk and low‐benefit treatments on patients. IRBs had to prioritize specific issues such as inclusion or exclusion criteria, participant compensation, and risk assessments for vulnerable patients to facilitate rapid research review and management of time (Table 3).

#### 3.3.2 Ethical issues after IRBs

Despite ethical review board approval, many studies deviated from their protocols due to circumstances during the study. To ensure transparency and efficiency, modifications to pre‐study documents, consent forms, study entry reports, conflict of interest, sponsorship, and side effects had to be reported to the ethics committee. This allowed for transparency and ensures that changes are made appropriately.^15^, ^39^

#### 3.3.3 Structure and process of IRB

Ethics committees faced the challenge of requiring a multidisciplinary team of experts in virology, infectious diseases, pharmaceuticals, and public health for quick and accurate document review.^28^ To address this, committees should prioritize investigator‐initiated trials from a public health perspective and expedite the review of academic trials that address important questions. Regulatory approval processes should be streamlined, redundancies in research design approval processes eliminated, and urgent public health trials facilitated. Experts in different fields can review these indicators.^39^ To expedite the approval of interventional studies, having only one national ethics committee review and approve studies is recommended. This approval should be accepted throughout the country without needing re‐approval by another hospital or city's ethics committee^20^ (Table 3).

### 3.4 Issues related to research conduction

#### 3.4.1 Clinical trials

Limited access to healthcare facilities and resources significantly impacted research during the COVID‐19 pandemic. Quarantine restrictions affected adherence to clinical study protocols, making it challenging to conduct studies, document procedures, and report adverse events and safety evaluations. This prevented the implementation of numerous clinical studies. Risk assessment was necessary to consider current risks and disadvantages when starting a new study or recruiting trial participants.^12^, ^42^

The pandemic significantly impacted clinical trials, particularly in cases where patient follow‐ups and randomization were halted, leading to economic losses. Many unnecessary experiments were stopped to prioritize the research with a greater benefit‐to‐harm ratio.^21^

Ethically speaking, exposing trial participants to risk is unacceptable if the study is not designed to provide valid results. Therefore, rigorous methodology should be implemented, including randomization, blinding, and placebo use, to enhance scientific validity and societal value. However, in severe epidemics, insisting on randomization can create a conflict between individual health and societal interests, precluding patients' autonomy in choosing their therapy.^66^ In a clinical trial conducted during the Ebola epidemic, Perez et al. recommended prioritizing individual patient interests over the reliability of trial methodology when faced with a high risk of death. In a pandemic scenario, a high number of seriously ill patients presenting simultaneously with a high mortality rate make it ethically unacceptable to randomly allocate patients from the same family or location to receive or not receive an experimental drug. Additionally, critically ill patients may find the randomization procedure difficult to understand.^67^ It would be unethical and impractical to conduct a randomized controlled trial (RCT) that asks patients or family members to consent to standard care when a potentially beneficial therapy is available. In the LOTUS China, an open‐label RCT, 31 patients' families (8.6%) did not provide consent. For the Ebola trial, investigators conducted one group open‐label non‐randomized trial, where all patients received Favipiravir with standardized care. The investigators used historical mortality data to define efficacy endpoints and a target mortality threshold a priori, which was valuable in deciding whether to stop or continue the trial and guide data analysis and interpretation. This approach could improve the utility of efficacy information from non‐randomized trials. The World Health Organization (WHO) has planned SOLIDARITY, a large global trial of four drugs—Remdesivir, Chloroquine and Hydroxychloroquine, Lopinavir‐Ritonavir, and lopinavir‐ritonavir plus interferon‐beta. Its simple design allowed physicians to recruit confirmed COVID‐19 cases after obtaining informed consent and administer any of the four available drugs as per randomization by the WHO.^68^, ^69^, ^70^

##### Patient enrolment

One of the problems in conducting research during the COVID‐19 pandemic was patient enrolment. VACCELERATE Volunteer Registry was one of the systems that facilitated the enrolment of patients into studies. VACCELERATE is a comprehensive and coordinated database for conducting and enrolling volunteers for Phase II and Phase III clinical trials. Moreover, this registry can also be expanded to test vaccines on humans in future health emergencies.^43^

The pandemic limitations urged new measures for retaining study participants and registering new participants. Strong communication and commitment to participants, creating technological capabilities for teleworking, visits, and delivery of study medication are essential in effectively retaining study participants and recruiting new participants during the COVID‐19 pandemic.^34^, ^35^, ^52^ Facilitating remote patient visits, motivation to perform procedures at the patient's home, permission to use healthcare facilities, direct distribution of the medicine to the patient's home by site personnel or sponsors, and extension of reimbursement to patients and caregivers are solutions that can facilitate the process of clinical studies in pandemic crisis.^20^, ^36^, ^45^, ^63^ Online platforms and social media were among the most practical strategies to reduce the imposed limitations.^29^ Simmons et al.^46^ replaced all in‐person parts of their clinical study using two key technology platforms: Study Pages (Yuzu Labs Public Benefit Corporation, 2022) and Pattern Health (Durham, NC). Recruitment and screening, consent, enrollment, randomization, data collection, blinding, adherence, and retention were performed with these platforms.

While recruiting study subjects can be difficult in typical circumstances, the COVID‐19 pandemic posed additional obstacles for individuals and children seeking to participate in pediatric nursing research. Skeens' study found that using social media to recruit a sample of parent‐child dyads during the COVID‐19 pandemic was an innovative technique.^51^ In addition, an original web‐based survey determined that social media was a successful and efficient technique for gathering data on COVID‐19 in a short period of time.^27^

##### Faster research dissemination

In response to COVID‐19, the research community has rapidly adopted a new way of disseminating research. However, unfortunately, the way in which research is being conducted has not changed. There has been an unprecedented surge of COVID‐19‐related preprints and peer‐reviewed publications. While preprint servers and faster peer review processes have clear merits, such as quicker dissemination of results, informing policies, and speeding up the R&D process for COVID‐19 therapeutics and vaccines, the quality of COVID‐19 research has been largely subpar. Many preprints, which are not peer‐reviewed, were rushed to dissemination without sufficient oversight, leading to potential inaccuracies and false claims.^31^

##### Employing virtual platforms

Limited face‐to‐face interactions during the pandemic significantly reduced the number of research visits, and study evaluations, requiring most research visits to be conducted remotely or via phone or video calls. For example, in drug effectiveness studies, by editing the protocols, the study medications could be mailed to the participants instead of in‐person deliveries.^7^, ^15^

The lack of experience regarding virtual platforms to implement clinical studies also affected the results. Lack of face‐to‐face communication, the reduction of interpersonal interaction between the researcher and the participants, and the accuracy of the acquired information were among the limitations that could cause bias in clinical studies.^39^

Each remote data collection method has its advantages and disadvantages that determine its feasibility and acceptability in certain settings. For example, when considering a mobile phone survey, IVR and SMS surveys are more affordable than CATI, but require participants to have high literacy levels. CATI, on the other hand, allows for the inclusion of individuals regardless of their literacy level and provides opportunities for researchers to encourage participation and clarify questions. In low‐ and middle‐income countries, where mobile phone ownership is widespread but access to smartphones and the internet is limited, mobile phone methods are more commonly used and are the focus of this commentary. However, few experts interviewed had implemented or planned online strategies due to their limited reach in certain low‐ and middle‐income countries. Some exceptions include online surveys designed for specific target groups, such as members of established professional associations and university students.^37^

#### 3.4.2 Epidemiologic studies

Epidemiological studies, like other studies, have been affected by COVID‐19. During recruitment and longitudinal assessments, epidemiologic studies are susceptible to refusals and losses of follow‐up. In face‐to‐face data collection, researchers adopt strategies such as changing the interviewer or contacting the participant on different days/times to mitigate this issue. However, researchers cannot pinpoint the number of people reached by internet‐based approaches. While some social media platforms, such as Instagram, allow publishers to see how many people were reached by posted advertisements, others, like WhatsApp, do not. Thus, it is not possible to calculate refusal/loss rates. However, sample size calculations should consider a certain percentage of losses and refusals. Therefore, sample size calculations should be conducted before data collection begins, and researchers should devise a recruitment strategy that allows them to reach the previously defined sample.^32^

#### 3.4.3 Data analysis

One of the essential component of clinical studies is statistical models methods.^22^ Statistical methods are necessary to prevent or minimize the risk of bias, a common threat in clinical and epidemiological studies. Obtaining appropriate clinical information from  patients with COVID‐19 in the city of Wuhan was only possible by the epidemiological data. Data Integration and cleaning from large multicenter hospitals are critical and require complex data management. Artificial intelligence (AI) and deep learning algorithms can be crucial in dealing with these challenges. AI and machine‐learning solutions could have a significant impact on fighting the disease. For instance, machine learning techniques have been used intensively in studying different conditions regarding protein analysis, forecasting, prediction, and paving the way towards vaccines and antivirals. An example of such a disease is the seasonal Flu. From this perspective, many AI approaches (including disease forecasting, surveillance, expected peak, and spread models) have been proposed and developed for several diseases, including the seasonal flu, which is relatively similar in its symptoms to COVID‐19.^64^

There was also a need for an international committee of statistical experts to decide on statistical methods during the COVID‐19 pandemic.^33^, ^71^ Additional measures were needed besides the usual strategies for conducting a clinical trial to deal with the mentioned challenges in a pandemic. The conditions of participation, measures needed to prevent infection, and the possibility of withdrawing from the study should be available before making decisions for participants at increased risk of infection.^7^, ^24^

#### 3.4.4 Research protocols and guidelines during a pandemic

During a pandemic, data security, patient satisfaction, and ethical statements, which are necessary in non‐pandemic situations, can be considered bureaucratic obstacles. However, rapid access to clinical data during epidemic circumstances requires special handling of these matters, which should be discussed nationally.^23^ Another statistical challenge during the COVID‐19 pandemic was that many clinical studies were not implemented according to written protocols due to the inability to blind, obtain a high sample size, and randomize. Therefore, statistical methods must be adapted to pandemic conditions. Data should be collected and analyzed in a standardized way, and statisticians are encouraged to develop appropriate analytical strategies for data collected from standardized protocols such as ISARIC and LEOSS. Rapid and valid information flow and reporting are crucial during a pandemic, and long‐lasting reporting guidelines may do more harm than good. Specific reporting guidelines are needed for pandemic settings.^23^

Another challenge was related to studies started before the pandemic that were affected by COVID‐19. Challenges included discontinuation of medication, withdrawal of a significant number of participants, deaths due to COVID‐19, and changes in study arms, which were not foreseen and affect study designs. Changing and updating the study protocol, continuing the investigation, and performing sensitivity analysis for missing data can be suitable solutions.^25^

#### 3.4.5 High‐risk populations

Research on the elderly population with chronic diseases posed another challenge. To prevent disruptions in research implementation for this population, patient registry systems, improved interactions with other institutions associated with the elderly, and improved study participation conditions such as transportation, health, and safety are necessary.^43^ COVID‐19 has also posed one of the biggest challenges for non‐COVID‐19 research on older people. The pandemic has made research challenging to conduct in practice and diverted the time and resources of investigators, funders, regulators, and delivery teams away from non‐COVID‐19 research. Survey data from the British Association of Stroke Physicians showed that most UK stroke research projects had been halted, and all responding sites had seen a substantial decrease in stroke research activity. The economic shock delivered by the pandemic is likely to lead to significant cuts to public and charity budgets worldwide, and it is unclear to what extent this will affect medical research. Even if medical research budgets are preserved, COVID‐19‐related research will likely compete with non‐COVID‐19 research for funding.^26^

Funding and financial sponsorship were other prominent issues during the COVID‐19 pandemic. Most funds were devoted to the treatment of patients and protective measures, leading to financial challenges that need to be resolved by organizations and institutions during crises and pandemics. To address this issue, a top‐down decision‐making mechanism was established in the European Union, where adequate funding was quickly provided through the Horizon Europe and ERA4Health budgets.^48^

## 4 DISCUSSION

### 4.1 Main findings

In this study, we reviewed fundamental challenges in conducting clinical research in the era of COVID‐19 pandemic. Individuals, communities, and societies are facing severe social, physical, and emotional challenges during the COVID‐19 pandemic. Decisions about conduct research using remote methods should consider the research burden and the risks associated with COVID‐19 to study participants.

### 4.2 Comaprison with previous studies

Remote data collection requires much effort from the study participants, who may need to use their own resources, such as a phone, internet access, and identifying a private space to participate in the study. On the other hand, remote methods may be preferable for study participants and eliminate the time and opportunity costs associated with travel to study sites. As with any research, the potential risks must be weighed against the benefits and the ethical imperative to continue the research to produce evidence useful for public health.^37^ Original studies also showed that remote data collection was an effective way to deal with the restrictions created by COVID‐19. Also, original studies determined that using technology like social media was an effective strategy for conducting research.^27^, ^51^

Key challenges in remote data collection encompass gathering diverse experiences in qualitative research, obtaining a representative sampling frame of the target population in quantitative research, and reaching out to more accessible populations.^63^, ^72^ While some of these challenges also exist in face‐to‐face research, the limited ability to personally recruit participants, whether at home, in a clinic, or any other locations, along with the reliance on mobile phones for recruitment, poses a specific challenge. This necessitates the exploration of alternative sampling methods for qualitative research, including purposive, snowball, and convenience sampling.

Purposive sampling aims to ensure diversity by considering key factors that are theorized to influence the experience. Recruitment can be facilitated through community‐based organizations, influential community leaders, neighborhood health committees, or established networks. Snowball sampling can be an effective approach for qualitative research; however, it is crucial to involve several initial participants who can then recruit others from within their own networks to achieve the desired diversity.^73^, ^74^ These sampling methods can also be used in quantitative research. Snowball sampling may be useful for online surveys shared via email or social media platforms,^75^ and a convenience sample can be employed through online social networking platforms.

Verbal consent (via phone or voice note) or written consent (via email, WhatsApp, or SMS) is accepted by some ethics committees because written informed consent becomes challenging or impossible during a pandemic. For mobile phone‐based research with adolescents, which requires parental consent, additional challenges arise in verifying the participant's age to determine the adolescent consent. Parents' satisfaction should be examined in line with adolescent satisfaction. For these reasons, verbal consent may be preferred over written consent, which can be recorded or performed in conjunction with written consent. Concise and simple language is required to convey complete information remotely while maintaining the strict ethical standards of face‐to‐face research. Consent should always be documented appropriately while protecting patient information and confidentiality. Documentation can take the form of a list of participants, stored on a password‐protected computer, who have consented to participate in various study components, which can also serve as a record for audit purposes.^37^

The privacy and safety of participants are crucial considerations when conducting research. In face‐to‐face studies, it is the responsibility of the researcher to establish and ensure privacy, and data collection must be halted if privacy is compromised. However, the onus is placed on the study participant in remote research to maintain their privacy. Nonetheless, establishing privacy can be challenging when participants share living spaces and have limited access to private areas and time. This becomes particularly significant in studies that examine sensitive topics like gender‐based violence, where compromised privacy can have harmful consequences.^76^

To address this issue, it is essential to inform participants about the potentially sensitive nature of the study at the beginning of data collection and encourage them to seek a private space. Strategies such as incorporating “passwords” or “exit buttons” can be implemented to mitigate risks. These mechanisms allow participants to verbally state or click on an option to indicate a breach of privacy.^76^ IVR and online surveys allow participants to complete surveys at a time and place of their choice, enabling them to establish privacy more effectively. Furthermore, these surveys can include a question asking respondents whether they completed the survey in private or in the presence of someone else, such as their child, parent/guardian, or friend.^37^

Data protection, including end‐to‐end encryption of phone calls and the security of platforms used to deliver online surveys and interview transcripts, is an additional privacy and confidentiality issue that needs to be addressed.^77^ In addition, researchers have the duty of care and should carefully consider safeguarding issues, particularly where COVID‐19 has affected the availability of support services. Information about online or telephone services must be available during the consent process. Specific referral protocols should be established, interviewers should be notified if certain responses may trigger automatic referrals, and follow‐up should be provided if safeguarding issues arise. As part of this protocol, researchers must establish a system to regularly check that these services remain operational.^42^

### 4.3 Strengths and limitations

This is the first review to study both the challenges and solutions of conducting clinical research during the COVID‐19 pandemic, providing a practical guide for researchers and policymakers in future similar pandemic conditions. However, this study had some limitations. We had to rely on primary studies, as there was not enough information about the challenges of conducting studies in all types of research. Additionally, the majority of the studies discussed in this article were in the form of editorials, highlighting the need for more rigorous studies to investigate the subject matter further.

Nevertheless, this study has proposed effective solutions that policymakers can consider for implementation in the context of decision‐making for addressing the ongoing pandemic and future crises. Although WHO has declared the end of the COVID‐19 pandemic,^65^ this review can still provide valuable information to achieve structured guidelines for researchers in future crises.

## 5 CONCLUSION

The study findings revealed significant challenges associated with conducting research during the COVID‐19 era. These challenges span various stages, ranging from research inception and study approval to patient enrollment and data analysis. Existing solutions must be adapted to the prevailing circumstances, highlighting the importance of enhancing the underlying research infrastructure to ensure continuity during times of crisis and pandemics. Numerous studies have proposed remote methods and electronic equipment as viable approaches to conduct research. However, the successful implementation of these methods relies on the availability of adequate infrastructure and adherence to country‐specific national and university policies.

## AUTHOR CONTRIBUTIONS

**Mahin Nomali**: Data curation; Formal analysis; Investigation; Validation; Writing—original draft. **Neda Mehrdad**: Conceptualization; Investigation; Supervision; Validation. **Mohammad Eghbal Heidari**: Data curation; Investigation; Writing—original draft. **Aryan Ayati**: Writing—original draft; Writing—review & editing. **Amirhossein Yadegar**: Writing—review & editing. **Moloud Payab**: Supervision; Validation. **Alireza Olyaeemanesh**: Data curation; Investigation. **Bagher Larijani**: Conceptualization; Project administration; Supervision; Validation.

## CONFLICT OF INTEREST STATEMENT

The authors declare no conflicts of interest.

## ETHICAL STATEMENT

All the authors declare that the present work has been carried out per the Journal's Practice Guidelines on Publishing Ethics and has been performed ethically and responsibly, with no research misconduct. The article has not been previously published and is not currently submitted elsewhere. The study proposal was passed by the ethical committee of the Iranian Academy of Medical Sciences (IAMS) (ID: IR.AMS.REC.1401.029).

## TRANSPARENCY STATEMENT

The lead author Bagher Larijani affirms that this manuscript is an honest, accurate, and transparent account of the study being reported; that no important aspects of the study have been omitted; and that any discrepancies from the study as planned (and, if relevant, registered) have been explained.

## Supporting information

Supporting information.

## Data Availability

All data associated with the article is available upon request.
