# Supplementary figures and images for: Challenges and solutions in clinical research during the COVID‐19 pandemic: A narrative review
Source: Health Sci Rep. 2023 Aug 6;6(8):e1482. doi: 10.1002/hsr2.1482 (PMC10404843; doi:10.1002/hsr2.1482)

**Supplementary:**


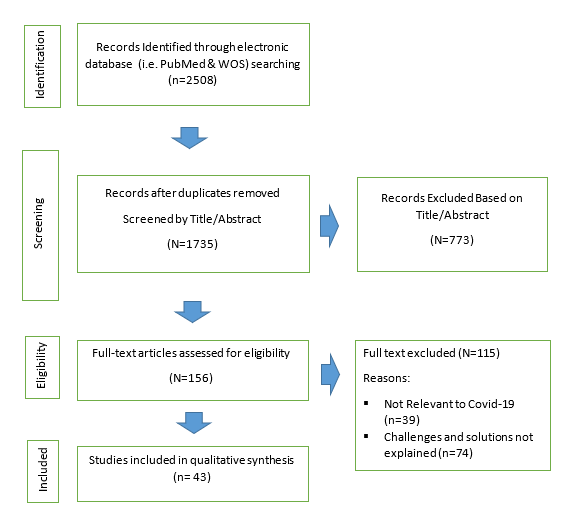


**Figure S1.** Flow diagram of the searching, screening, and selecting process

Supplement: Supplementary file 1 — Supporting information. [file HSR2-6-e1482-s001.docx]
